# An international multicenter experience with the Senhance^®^ Surgical System in pediatric surgery: Analysis of the first 150 pediatric procedures

**DOI:** 10.1007/s11701-026-03232-9

**Published:** 2026-02-23

**Authors:** Rianne E. M. Killaars, Daniel L. Widmann, Ruben G. J. Visschers, Hamit Cakir, Marc Dirix, Olivier P. F. Theeuws, Dianne J. H. Dinjens, Oliver Muensterer, Jan Gödeke, Wim G. van Gemert

**Affiliations:** 1 Department of Pediatric Surgery, MosaKids Children’s Hospital of the Maastricht University Medical Center+ (MUMC+), P. Debyelaan 25, Maastricht, 6229 HX The Netherlands; 2 European Consortium of Pediatric Surgery (Maastricht UMC+, Uniklinik Aachen, Clinique CHC MontLégia Liège), P. Debyelaan 25, Maastricht, 6229 HX The Netherlands; 3 Research Institute of Nutrition and Translational Research in Metabolism (NUTRIM), Universiteitssingel 40, 6229 ER Maastricht, The Netherlands; 4 Department of Pediatric Surgery at the Dr. von Hauner Children’s Hospital of The Ludwig Maximilian University (LMU) University Medical Center of Munich, Lindwurmstrasse 2a, 80337 Munich, Germany

**Keywords:** Pediatric surgery, Children, Infants, Robotic-assisted surgery, Senhance Surgical System, Minimally invasive surgery

## Abstract

Our previous studies indicated that the Senhance^®^ Surgical System (SSS^®^) is safe and feasible for a wide spectrum of abdominal indications in children, with results comparable to conventional laparoscopy. This multicenter cohort study represents the largest published series of pediatric Robotic-Assisted Surgery (RAS) procedures performed with the SSS^®^ to date and complements the existing evidence, which is currently based on single-center studies. A total of 152 pediatric patients underwent a variety of RAS procedures (including upper abdominal-, lower abdominal-, and thoracic procedures) using the SSS^®^ between 2020 and 2025 at two European tertiary referral centers and were consecutively enrolled in the study and analyzed. Of these, 23 children (15%) were younger than 1 year old and/or weighed less than 10 kg at the time of surgery. Conversion from RAS to conventional laparoscopy occurred in 17 cases (11%), with a significantly lower rate observed in lower abdominal procedures, and most often due to suboptimal trocar placement. Only one case was converted to an open procedure, with no conversions in the small children subgroup. Intraoperative- and postoperative complication rates were low, regardless of procedure type or patient age. Mean docking time decreased progressively over the course of the study (p = < 0.001). Utilization of SSS^®^ for RAS is safe and feasible across the entire pediatric spectrum of surgical procedures and patients, including infants younger than one year and children weighing less than ten kilograms. With increasing experience, the SSS^®^ has significant potential to advance minimally invasive surgery in children.

## Introduction

Over the past few decades, surgical practice has undergone a remarkable shift from open surgery to minimally invasive surgery (MIS) [1]. Laparoscopic surgery is performed through small incisions and within a more targeted surgical field. By minimizing manipulation of the peritoneum and surrounding structures, this method decreases the risk of abdominal adhesions [2]. It is also associated with other favorable postoperative outcomes, including fewer complications, less pain after surgery, better cosmetic results and shorter hospital stays [3]. As a result, laparoscopic surgery has become widely accepted across a broad range of surgical procedures. Pediatric surgery is sometimes late to adapt the implementation of new techniques and treatments, which is certainly true when it comes to MIS. Nevertheless, MIS has demonstrated superiority over open surgery, even in pediatric surgical practice [3–7]. Children, tend to recover faster and resume normal physical activities earlier following laparoscopic procedures when compared to adults [3, 4].

Robotic-Assisted Surgery (RAS) represents a further refinement of the laparoscopic technique. While aiming to keep the benefits of laparoscopy, RAS also addresses its inherent limitations. RAS improves surgical accuracy by offering a three-dimensional (3D) steady view instead of a two-dimensional (2D) view, using articulating instruments, eliminating hand tremors and unnecessary movements, and providing a more ergonomic, comfortable, seated operating position [8–10]. As a result, the use of RAS has seen significant uptake, supported by technological progress and growing clinical experience.

RAS in pediatric practice also faces significant challenges. Most robotic systems are designed for adult anatomy, making their use in smaller pediatric patients technically more difficult [11]. Essential pediatric requirements, like open communication, unobstructed access to the patient, and adaptable trocar positions, are hindered by the size and rigidity of adult robotic platforms. To improve precision and reduce tissue strain, the fulcrum point must be closer to the surgical site, which necessitates the use of shorter, smaller instruments [12].However, RAS can offer advantages which are especially suited for children. Pediatric anatomy and physiology, including anatomical structures, body size, organ position, and physiological functions alter according to age. In robotic systems with a modular system, the trocar positions can be adapted and optimized based on these characteristics. The improved 3D view of RAS may help in better identifying and preserving small critical structures [8]. Smaller instruments improve access and dexterity in a very small operating field and tremor filtration, allowing for more delicate suturing and dissection in the very small anatomical spaces of children and newborns [13, 14]. RAS offers enhanced precision, faster recovery, and less pain, being especially significant factors in children, considering their potential influence on growth and quality of life [15, 16].

To fully express the potential of robotic surgery in pediatrics, the robotic systems must be adapted. The Senhance^®^ Surgical System (SSS^®^) is designed specifically for pediatric use, offering 3-mm and 5-mm instruments that have been proven usable across a wide range of ages [17, 18]. Combined with trocar placement options that can be adjusted to the child’s body size and proportions, the system has been described as suitable even for very small pediatric patients [12, 19]. Additionally, the reusability of the instruments is noteworthy, as it may help address one of the main barriers to RAS in pediatrics: cost-effectiveness [10].

Our previous studies indicated that the SSS^®^ in children is safe and feasible for abdominal indications, with results comparable to traditional laparoscopic surgery [18, 20, 21]. However, most current pediatric RAS is based on single-center studies, and multi-center analyses remain limited. This study aimed to evaluate the outcomes of pediatric RAS using the SSS^®^ at two major European academic pediatric surgery centers, focusing on intraoperative and postoperative results and possible factors responsible for the outcome variability.

## Materials and methods

### Study setting

This retrospective, observational cohort study was conducted at two European tertiary referral centers: MosaKids Children’s Hospital (of Maastricht University Medical Center + (MUMC +), the Netherlands) and Dr. von Hauner Children’s Hospital (of The Ludwig Maximilian University (LMU) University Medical Center of Munich, Germany). Both centers have significant experience in pediatric minimally invasive surgery and were among the early adopters of the SSS^®^ in children. The analysis included all consecutive pediatric surgical procedures using the SSS^®^ performed from January 2020 to April 2025 in both centers.

### Study population

During the study period from 2020 to 2025, approximately 1800 pediatric laparoscopic and 55 thoracoscopic procedures were performed across the two participating centers. Of these, 152 procedures (1:12 cases) were performed robotic-assisted using the SSS^®^. All children indicated for laparoscopic surgery were considered eligible for RAS and, therefore, for inclusion in the study. The final surgical approach was determined by surgeon judgment, patient characteristics, and logistical factors. No other robotic surgical platforms were used for children at either institution during the study period.

All consecutive patients under 18 years old who underwent RAS using SSS^®^ during the study period, and for whom informed consent was obtained, were enrolled. No exclusion criteria were applied in order to capture all events. Patient demographics, including age, sex, weight, and surgical indication, were collected. Procedures were classified according to anatomical region (upper abdominal, lower abdominal, and thoracic) and patient size, determined by age and weight, to assess feasibility and outcomes across a wide pediatric spectrum.

### Data and clinical outcomes

Intraoperative data were collected during the RAS procedures, and pre- and postoperative data were retrospectively extracted from electronic medical records and operative reports in each center. Variables included patient demographics, procedure type, instrument size, operative time (skin-to-skin), robot docking time, intraoperative events (such as conversion from RAS to laparoscopy or open surgery), complications, and postoperative outcomes like complications, hospital stays, readmission, and reoperation within 30 days.

The data reflects all RAS procedures using the SSS^®^ in the MosaKids Children’s Hospital (of MUMC +) in Maastricht and the von Hauner Children’s Hospital (of LMU University Medical Center) in Munich. This study evaluated the effectiveness of RAS across different age groups, weight categories, and anatomical regions using the SSS^®^ in children by analyzing the primary outcomes: the rate of unplanned conversion and the procedure completion rate. Secondary outcomes included intraoperative and postoperative complications, total operative time, docking time, and length of postoperative hospital stay. The safety of RAS in all anatomical regions in children of different ages using the SSS^®^ was evaluated through analysis of intraoperative and postoperative complications (graded according to the Clavien-Madadi classification).

### Surgical team and procedure specifics

In Maastricht, RAS procedures were performed by five pediatric surgeons who rotated through the cases, all with ample prior experience in minimally invasive surgery and RAS. In Munich, two senior pediatric surgeons performed all procedures. In both locations procedures were not performed by fellows or residents as primary operators. Surgeon experience and team continuity were maintained throughout the study to minimize variability in operative time, conversion rates, and learning-curve outcomes.

Instrument size selection (3-mm versus 5-mm) was primarily determined by patient size and anatomical considerations. 3-mm Instruments were generally preferred in smaller children (weighing less than ten kilograms) and in procedures with limited working space, to optimize instrument maneuverability and minimize abdominal wall trauma. Surgeon preference was considered when either instrument size was feasible.

### Statistical analysis

Statistical analyses were performed using IBM SPSS Statistics, version 29.0 (IBM Corp., Armonk, NY, USA). Continuous variables are reported as mean standard deviation (SD) when normally distributed (assessed with the Shapiro–Wilk test). Categorical variables are summarized as a number (percentage). All tests were two-sided, and a p-value of < 0.05 was considered statistically significant.

A priori, children were dichotomized into “small children” (younger than 1 year old and/or weighing less than 10 kg) and “older children” (all others). Differences in conversion rates, intraoperative and postoperative complications, readmission, and re-operation between these two groups were assessed using Pearson’s chi-square test (or Fisher’s exact test when expected cell counts were less than 5). For continuous outcomes, an independent samples t-test was used; if normality was questionable, the Mann–Whitney U-test was applied.

To assess procedure-related outcome differences, children were categorized into three groups prior to analysis: 1) upper abdominal procedures (UAP), 2) lower abdominal procedures (LAP), and 3) thoracic procedures (TP). Outcomes across these groups were compared using Chi-square tests for categorical data and one-way ANOVA for continuous data. Tukey-adjusted pairwise comparisons (post-hoc test) were used to identify specific group differences.

For learning-curve assessment, cases were numbered chronologically (1–152). Because the two centers adopted the system at different times, docking time analyses focused on the center Maastricht UMC + with having the most cases done (case 1–112). Operating- and docking times between periods were compared with one-way ANOVA.

## Results

### Procedures and pediatric patient characteristics

A total of 152 pediatric patients underwent a RAS procedure using the SSS^®^ during the study period. All patients who underwent surgery between January 2020 and April 2025 at Maastricht or Munich were successfully included in the analysis and completed follow-up.

The study group consisted of 84 male (55%) and 68 female (45%) pediatric patients (aged 0 to 17 years), with a mean age of 8.3 years (± 5.7) and a mean body weight of 32 kg (± 24). Of these, 23 children (15%) were younger than 1 year old and/or weighed less than 10 kg at the time of surgery. The youngest child to undergo RAS was fifteen days old and underwent surgery for an intra-abdominal and intrapelvic portion of an Altman type 4 sacrococcygeal teratoma.

Patients underwent a variety of procedures, categorized as upper abdominal procedures (UAP, n = 69; 46%), lower abdominal procedures (LAP, n = 78; 51%), or thoracic procedures (TP, n = 5; 3%). The most frequently performed procedures included inguinal hernia repair (IHR, n = 45), Nissen fundoplication (NF, n = 40), and cholecystectomy (n = 17). An overview of the procedures, numbers performed, patient age and weight, and the instrument sizes used is provided in Table 1.

The 3-mm instruments were used in 36 out of 152 cases, with significantly higher usage in patients weighing less than 10 kg or being younger than 1 year old (n = 15; 65%) compared to larger patients (n = 21; 16%) (p < 0.001). As shown in Table 1, 3-mm instruments were most frequently used in lower abdominal procedures, corresponding to the subgroup with the highest proportion of smaller children.

**Table 1:** An overview of the Robotic-Assisted Surgery (RAS) procedures performed with the Senhance^®^ Surgical System (SSS^®^) at MosaKids Children’s Hospital (Maastricht University Medical Center +, the Netherlands) and Dr. von Hauner Children’s Hospital (of The Ludwig Maximilian University University Medical Center of Munich, Germany).

Robotic-assisted surgery procedures	Number performed (%)	Mean age^1^	Mean weight^2^	F/M ratio^3^	Number small children (%)^4^	3 mm/5 mm-instrument ratio^5^
**Upper abdominal procedures (UAP)**	**69 (46)**	9.9 (± 5.5)	39 (± 27)	33/36	8 (12%)	11/58
Nissen fundoplication Cholecystectomy Heller-Dor procedure Resection of esophageal duplication cyst Splenectomy/resection of splenic cyst Esophagectomy Resection choledochal cysts	40 17 3 2 5 1 1					
**Lower abdominal procedures (LAP)**	**78 (51)**	6.8 (± 5.3)	26 (± 19)	33/45	13 (17%)	23/55
Inguinal hernia repair Appendectomy Ileocecal resection Ladd’s procedure and appendectomy Caecostomy Proctocolectomy (with IPAA) Ileostomy Teratoma extirpation Subtotal colectomy Pull-through procedure (Hirschsprung) Pyeloplasty Nephrectomy with urethrectomy Urachal resection Paraganglioma resection left bladder	45 6 5 4 3 1 1 2 1 1 6 1 1 1					
**Thoracic procedures (TP)**	**5 (3)**	9.5 (± 8.1)	37 (± 27)	2/3	3 (60%)	2/3
Lung sequester resection	5					
**Total procedures**	**152 (100)**	8.3 (± 5.7)	32 (± 24)	68/84	23 (15%)	36/116

^1^Age at time of surgery, in years

^2^Body weight at time of surgery, in kilograms

^3^M = male, F = female

^4^Small children were defined as those younger than 1 year old or weighing less than 10 kg

^5^Size of the (work)instruments of the Senhance® Surgical System used during the procedure

### Intraoperative outcomes

#### Operative time and docking time

**Fig. 1:**
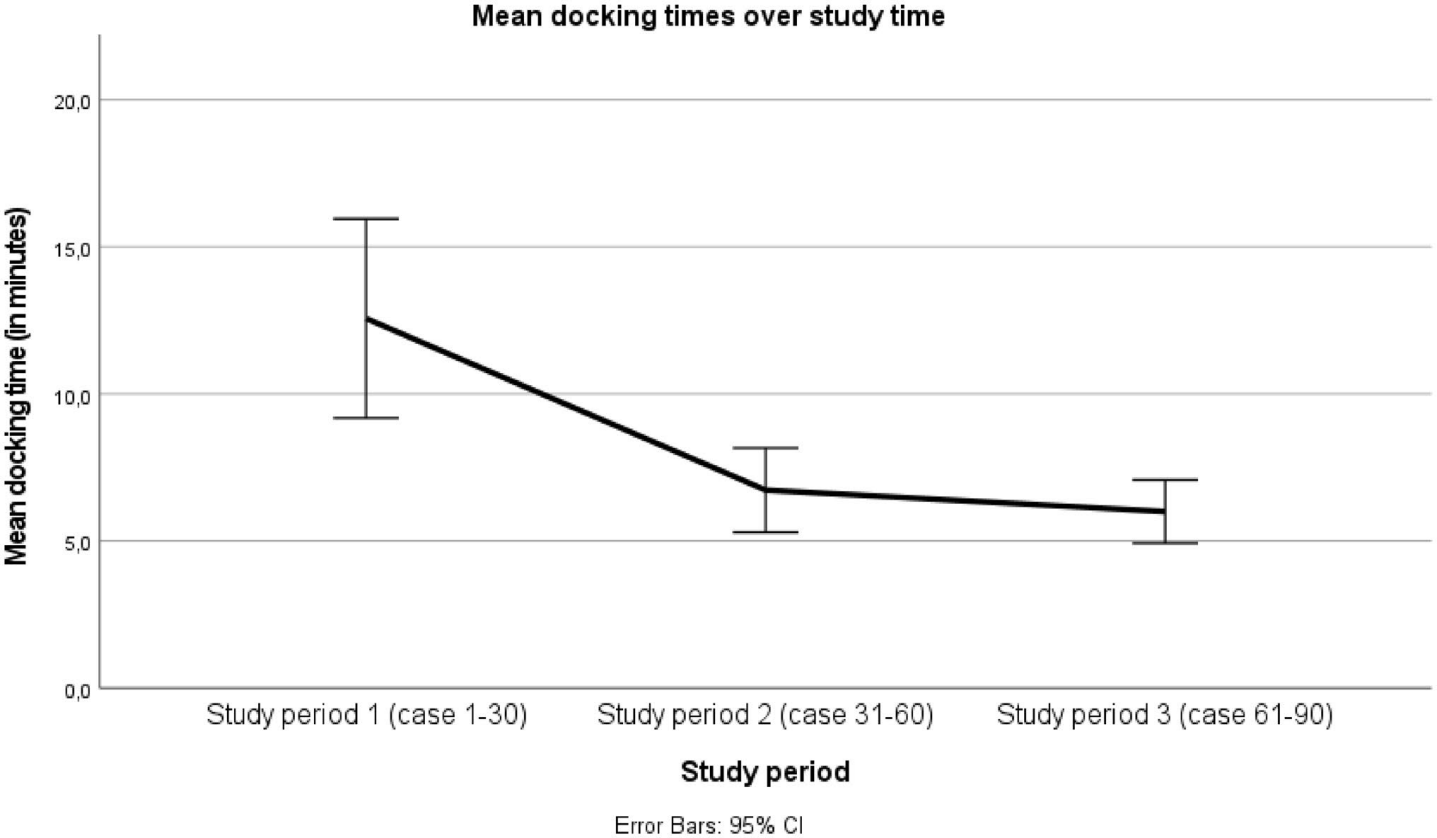
Mean docking times of the robot during the Robotic-Assisted Surgery (RAS) cases performed in MosaKids Children’s Hospital (Maastricht University Medical Center +, the Netherlands), for time period 1 (case 1–30), time period 2 (case 31–60), and time period 3 (case 61–90). There was a significant decrease in the mean docking time during period 2 and period 3 compared to the initial period 1 (p = < 0.001)

The mean operative time for all RAS procedures was 136 min (± 87). Trends towards shorter operative times over time were observed in all three of the most common surgeries (Nissen fundoplication, cholecystectomy and inguinal hernia repair) with learning curves differentiating between surgeries of different complexity (Table 2). The mean docking time of the robotic system across all the 152 procedures was 9 min (± 6). During the RAS cases performed at the MosaKids Children’s Hospital (Maastricht University Medical Center +, the Netherlands), a significantly longer docking time was observed during the initial learning phase (period 1, case 1–30: 13 ± 8 min). The mean docking time decreased progressively in period 2 and period 3 compared with period 1 (p = < 0.001) (Fig. 1).

**Table 2:** Learning-curve analysis showing decreases in mean operative time over consecutive study time periods for selected procedures

Specific procedure (number of cases MUMC +/number of cases LMU)	Study time period	Mean operative time (in minutes)
Nissen fundoplication (25/15)	T1	177
	T2	149
	T3	138
Cholecystectomy (9/6)	T1	133
	T2	103
	T3	81
Inguinal hernia repair (45/0)	T1	93
	T2	65
	T3	66

MUMC+ = Maastricht University Medical Center+; LMU =The Ludwig Maximilian University University Medical Center of Munich.Cases were divided into three consecutive time periods (T1–T3) based on case order across participating centers. For Nissen fundoplication and cholecystectomy, cases from both centers were combined; for inguinal hernia repair, cases were derived from a single center (MUMC+)

#### Conversions

Conversion from RAS to conventional laparoscopy occurred in 17 of 152 cases (11%), with a significantly lower rate seen in LAP (n = 5/78; 6%) compared to UAP (n = 12/69; 17%) and TP (n = 2/5; 40%) (p = 0.022, with standardized residuals of –1.5 (LAP), 1.1 (UAP), and 1.7 (TP), indicating fewer-than-expected conversions in LAP and more-than-expected in UAP and TP). Most conversions to laparoscopy occurred during the first study period compared with the second study period (10 vs 7; see Table 3), which descriptively suggests a higher conversion rate during the initial learning phase.

**Table 3:** Clinical characteristics and outcomes of pediatric patients who underwent a Robotic-Assisted Surgery (RAS) procedure using the Senhance^®^ Surgical System (SSS^®^) (n = 152)

	Total procedures (n = 152)	UAP (n = 69)	LAP (n = 78)	TP (n = 5)	*P* value^1^
**Clinical characteristics**					
Sex in no. (%)					
Male Female	84 (55) 68 (45)	36 (52) 33 (48)	45 (58) 33 (42)	3 (60) 2 (40)	0.833
Age at time of surgery, mean in y (SD)	8.3 (± 5.7)	9.9 (± 5.5)	6.8 (± 5.3)	9.5 (± 8.1)	0.003*
Body weight at time of surgery, mean in kg (SD)	32 (± 24)	39 (± 27)	26 (± 19)	37 (± 27)	0.004**
**Intraoperative**					
Conversion to conventional laparoscopy, no. (%) Period 1 Period 2	17 (11) 10 7	12 (17)	5 (6)	2 (40)	0.022***
Conversion to open procedure, no. (%) Period 1 Period 2	1 (0.7) 0 1	0 (0)	0 (0)	1 (20)	0.033****
Operative time^2^, mean in minutes (SD)	136 (± 87)	142 (± 53)	129 (± 105)	167 (± 152)	0.487
Docking time of robot^3^, mean in minutes (SD) Period 1 (case 1–30 MUMC +) Period 2 (case 31–60 MUMC +) Period 3 (case 61–90 MUMC +)	9 (± 6) 13 (± 8) 7 (± 4) 6 (± 2)	7 (± 4)	10 (± 7)		
Intraoperative complications, no. (%) Period 1 Period 2	4 (3) 2 2	4 (6)	0 (0)	0 (0)	0.076
**Postoperative**					
Postoperative hospital stays^4^, mean in days (SD)	3 (± 5)	4 (± 6)	3 (± 5)	4 (± 5)	0.409
Postoperative complications^5^, no. (%) Period 1 Period 2	19 (13) 14 5	6 (9)	12 (15)	1 (20)	0.309
Readmission through 30 days, no. (%)	7 (5)	4 (6)	2 (3)	1 (20)	0.140
Reintervention, no. (%)	7 (5)	4 (6)	2 (3)	1 (20)	0.140
Mortality, no. (%)	0 (0)	0 (0)	0 (0)	0 (0)	
Clavien-Madadi Classification for complications [22], no					
1 2 3 3a 3b 4a 4b 5	2 7 1 1 8 1 1 0				
Total number of complications, no. (%)	23 (15)	10 (15)	12 (15)	1 (20)	

UAP = Upper Abdominal Procedures; LAP = Lower Abdominal Procedures; TP = Thoracic Procedures; y = years, kg = kilograms; no. = number. MUMC+ = Maastricht University Medical Center+; LMU =The Ludwig Maximilian University University Medical Center of Munich. For the analysis of learning-curve effects related to conversions and complications, the study period was divided into two phases: period 1 (cases 1–56 at MUMC and cases 1–20 at LMU) and period 2 (cases 57–112 at MUMC and cases 21–40 at LMU). Docking time was analyzed separately using three consecutive time periods and was restricted to single-center data (MUMC).^1^Outcomes across these groups were compared using Chi-square tests for categorical data and one-way ANOVA for continuous data. Tukey-adjusted pairwise comparisons (post-hoc test) were used to identify specific group differences. A p-value of less than 0.05 was deemed statistically significant. ^2^Operative time refers to the duration of the surgery, from the initial skin incision to the final wound closure. ^3^ Docking time is defined as the period required to position the robot and ensure the instruments are correctly aligned. ^4^ Postoperative hospital stay is calculated based on the number of nights spent in the hospital after the surgery (i.e., discharge on the same day is considered 0 nights, while discharge the next day counts as 1 night). ^5^Postoperative complications are defined as any issues that arise within 30 days following the surgery. * When comparing the group UAP to the LAP group separately (post-hoc test), the p-value is 0.002. ** When comparing the group UAP to the LAP group separately (post-hoc test), the p-value is 0.003. *** One-way ANOVA showed a significant difference among groups, with standardized residuals at post-hoc test of 1.1 (UAP), –1.5 (LAP), and 1.7 (TP). **** One-way ANOVA showed a significant difference among groups, with standardized residuals at post-hoc test of –0.7 (UAP), –0.7 (LAP), and 5.3 (TP)

Reasons for conversion from RAS to laparoscopy included limited instrument motion and/or robotic arm collisions (10 cases), most often related to suboptimal trocar placement. Additional reasons were a defective instrument (3 cases), poor visualization or concern for iatrogenic injury (3 cases), and the need to use a CoolSeal device during thoracoscopic sequestration resection (1 case).

Only one of the 152 RAS cases (0.7%) were converted to an open procedure. This was a thoracoscopic procedure where dense adhesions of the vascular structures and fibrotic lung parenchyma necessitated open access to allow precise palpatory identification and exposure for surgical safety. Overall, 88% of the RAS cases completed successfully.

#### Intraoperative complications

Intraoperative complications occurred in 4 of the 152 cases (3%), all within the UAP group (6%), although this difference was not statistically significant (p = 0.076). In the UAP group, the complications included: a 0.9-year-old (10 kg) undergoing a Robotic-Assisted Nissen Fundoplication (RNF) with conversion to laparoscopy for a small suspected iatrogenic esophageal wall injury (no perforation, preventively sutured); two readmissions for acute abdominal pathology due to an intraoperative complication (a 3-year old child after RNF with a thermal gastric perforation, and an 11-year old child after robotic-assisted laparoscopic cholecystectomy with cystic duct leakage); and a robotic-assisted splenectomy converted to laparoscopy due to bleeding during vessel dissection in a 17-year old girl.

### Postoperative outcomes

The mean postoperative hospital stay until discharge was 3 days (±5), with no significant difference among procedure types (UAP: 4 ± 6 days; LAP: 3 ± 5; TP: 4 ± 5; p = 0.409). There were 7 readmissions (5%) within 30 days after discharge. Overall, 19 patients (13%) experienced postoperative complications, of which 7 patients (5%) required reintervention. Three of these reinterventions concerned the intraoperative complications (as noted above) that became apparent postoperatively. Other postoperative complications included thoracic drain placement after an apical pneumothorax following thoracic surgery, endoscopic dilatation after Nissen fundoplication, and two relaparoscopies for peritonitis, in which abdominal rinsing alone was performed. Wound problems (such as dehiscence, infection, seroma, and hematoma) were seen in 4 cases. In all, 5-mm instruments were used during RAS procedure. All patients with postoperative complications recovered completely. No intra- or postoperative deaths were reported. The highest incidence of postoperative complications was observed in the first study period (see Table 3).

### Outcomes by procedure group

Differences in outcomes were observed among the three anatomical based procedure groups. The rate of conversion from RAS to conventional laparoscopy was significantly lower in LAP (n = 5/78; 6%) compared to UAP (n = 12/69; 17%) and the highest conversion rate in TP (n = 2/5; 40%) (p = 0.022, with standardized residuals of –1.5 (LAP), 1.1 (UAP), and 1.7 (TP), indicating fewer-than-expected conversions in LAP and more-than-expected in UAP and TP). Intraoperative complications occurred exclusively in UAP (6%), although this did not reach statistical significance (p = 0.076). Postoperative complications, reinterventions, and readmissions showed no significant differences between procedure groups (p > 0.05), although the absolute rates were slightly higher in TP (20%). Operative times and postoperative hospital stay durations were comparable across groups (p > 0.4).

### Small children

A subgroup analysis was performed to compare pediatric patients being younger than 1 year old and/or weighing less than 10 kg (referred to as small children, n = 23) to those being older than 1 year or weighing over 10 kg (referred to as older children, n = 129) to assess the feasibility and safety of RAS in smaller infants. In this study, a total of 23 children were under 1 year old or weighed less than 10 kg and underwent a RAS procedure using the SSS^®^. Of all the 23 RAS cases in small children, 91% completed successfully. This small children group had a mean age of 0.8 years (± 0.4) at time of surgery, which was significantly lower than the mean age of 10 years (± 5) in the older children group. The youngest child operated on using the SSS^®^ was 15 days old (3.8 kg), and the lightest child operated on weighed 3.8 kg. The conversion rate from RAS to laparoscopy in small children was 9% (n = 2/23), compared to a conversion rate of 13% (n = 17/129) in older children (p = 0.740). Conversions from RAS to laparoscopy in small children occurred due to limited instrument mobility in one case and the requirement for additional visualization in the other. None of the 23 RAS cases (0%) in small children were converted to an open procedure, compared to one case (1%) in the older children group. Among the group of small children, the intraoperative complication rate was 4% (n = 1) and the postoperative complication rate was 9% (n = 2). The rates did not differ significantly from the older children group (p > 0.05). As expected, the use of 3 mm instruments was significantly more frequent in small children (65% vs 16.3%, p < 0.001). Additionally, in the smaller children group, docking times were significantly shorter for upper abdominal procedures (6.2 vs 9.0 min, p = 0.010) and thoracic procedures (6.0 vs 11.0 min, p = 0.002). At the same time, overall operative times were not significantly different (Table 4).

**Table 4:** Clinical characteristics and outcomes differences between “small children” (younger than 1 year old and/or weighing less than 10 kg) and “older children” (all others) who underwent a Robotic-Assisted Surgery (RAS) procedure using the Senhance® Surgical System (SSS^®^)

	Small children (< 1 y OR < 10 kg; n = 23)	Older children (> 1 y AND > 10 kg; n = 129)	Mean difference (95% CI)	*P* value^1^
**Clinical characteristics**				
Sex in no. (%)				
Male Female	18 (78) 5 (22)	66 (51) 63 (49)		
Age at time of surgery, mean in y (SD)	0.8 (± 0.4)	10 (± 5)	–9 (–10 to –8)	< 0.001
Body weight at time of surgery, mean in kg (SD)	8 (± 2)	36 (± 23)	–28 (–33 to –24)	< 0.001
Procedure, no. (%)				
UAP LAP TP	8 (35) 12 (57) 2 (9)	61 (47) 65 (50) 3 (2)		
**Intraoperative**				
Conversion to conventional laparoscopy, no. (%)	2 (9)	17 (13)		0.740
Conversion to open procedure, no. (%)	0 (0)	1 (1)		1.000
Operative time^2^, mean in minutes (SD) UAP LAP	124 (± 61) 155 (± 42) 111 (± 68)	138 (± 91) 140 (± 55) 132 (± 111)	–14 (–52 to 25 15 (–25 to 55) –21 (–85 to 43)	0.489 0.455 0.507
Docking time of robot^3^, mean in minutes (SD) UAP LAP	6 (± 2) 7 (± 2) 6 (± 1)	9 (± 6) 7 (± 4) 11 (± 7)	–3 (–5 to –1) –0.3 (–7 to –6) –5 (–8 to –2)	0.010 0.933 0.002
Intraoperative complications, no. (%)	1 (4)	3 (2)		0.485
**Postoperative**				
Postoperative hospital stays^4^, mean in days (SD)	2 (± 2)	3 (± 6)	–0.9 (–3.2 to 1.4)	0.441
Postoperative complications^5^, no. (%)	2 (9)	17 (13)		0.740
Readmission through 30 days, no. (%)	1 (4)	6 (5)		1.000
Reintervention, no. (%)	2 (9)	5 (4)		0.286
Mortality, no. (%)	0 (0)	0 (0)		
Use of 3-mm instruments, no. (%)	15 (65)	21 (16)		< 0.001

UAP = Upper Abdominal Procedures; LAP = Lower Abdominal Procedures; TP = Thoracic Procedures; y = years, kg = kilograms. ^1^Outcomes across these groups were compared using Chi-square tests for categorical data and one-way ANOVA for continuous data. Tukey-adjusted pairwise comparisons (post-hoc test) were used to identify specific group differences. A p-value of less than 0.05 was deemed statistically significant. ^2^Operative time refers to the duration of the surgery, from the initial skin incision to the final wound closure. ^3^Docking time is defined as the period required to position the robot and ensure the instruments are correctly aligned. ^4^Postoperative hospital stay is calculated based on the number of nights spent in the hospital after the surgery (i.e., discharge on the same day is considered 0 nights, while discharge the next day counts as 1 night). ^5^Postoperative complications are defined as any issues that arise within 30 days following the surgery

A priori, children were dichotomized into “small children” (younger than 1 year old and/or weighing less than 10 kilograms) and “older children” (all others). Differences in conversion rates, intraoperative and postoperative complications, readmission, and re-operation between these two groups were assessed using Pearson’s chi-square test (or Fisher’s exact test when expected cell counts were less than 5). For continuous outcomes, an independent samples t-test was used; if normality was questionable, the Mann-Whitney U-test was applied

## Discussion

The results of this multicenter cohort study confirm that RAS using the SSS^®^ is safe and feasible across the entire pediatric spectrum of surgical abdominal and thoracic procedures and patients, including infants younger than one year and children weighing less than then kilograms. With a conversion rate from RAS to conventional laparoscopy of 11%, only one conversion from RAS to open surgery, an intraoperative complication rate of 3%, and a postoperative complication rate of 13% without mortality, overall outcomes were favorable. The overall completion rate of the 152 RAS cases was 88%. This cohort represents the largest published series of pediatric RAS procedures performed with the SSS^®^ to date and complements the existing evidence, which has thus far been limited to smaller, single-center case series [10, 15, 17, 22, 23].

Compared to previous reports on SSS^®^, our study confirms feasibility and safety even in more complex procedures and very small patients. Earlier studies with the Da Vinci^®^ Surgical System (DVSS^®^) in pediatric surgery reported similar complication and conversion rates; however, they lacked key pediatric-specific adaptations such as 3-mm instruments or flexible trocar positioning [24]. These technical features represent distinct advantages of the SSS^®^ when operating in small body cavities.

Not all procedures benefit equally from RAS. While simple standard operations such as appendectomy may continue to be more efficiently performed laparoscopically, robotic systems offer advantages in more complex procedures such as fundoplication, choledochal cyst resection, or reconstructive surgery. In these settings, ergonomic benefits, tremor reduction, and 3D-visualization may translate into higher surgical precision and potentially improved long-term outcomes. For surgeons, the ergonomic seated position also provides significant advantages, particularly during long and technically demanding operations. In the long term this could prevent the well-known back- and neck problems seen in the surgical population performing intensive laparoscopic procedures for a longer time [25].

Analysis of docking times demonstrated a clear reduction over the course of the study, indicating a distinct learning curve. The assessment of conversion cases further supports this finding: most conversion occurred early in the study and were primarily due to technical issues such as suboptimal trocar placement or external arm collisions. With increasing experience, these difficulties were mitigated. Practical adjustments, particularly regarding trocar placements and patient positioning, proved critical for successful implementation.

Although a direct comparison with conventional laparoscopy of the operative times was not feasible in this study due to heterogeneous data, RAS procedures may have required longer operative times especially during the early phase of robot adoption. The learning curve, regarding optimal set-up and intraoperative repositioning of robotic arms, contributes to longer operative times specifically during the initial RAS cases [18, 20].

A major contribution of this study is the demonstration that RAS can be safely performed even in very small children and infants. Despite theoretical concerns regarding the limited intra-abdominal working space and fragile anatomy, subgroup analysis revealed no increased complication or conversion rates in children weighing less than 10 kg. Also, the non-occurrence of conversion to an open approach shows that RAS is feasible in these small children. The frequent use of 3-mm instruments in lower abdominal procedures reflects the smaller size of the patients in these cases, highlighting the importance of instrument selection in pediatric robotic surgery. On the contrary, procedures were successfully performed using 3-mm instruments, which are not available with other conventional robotic systems. Interestingly, shorter docking times were observed in smaller children, potentially explained by their smaller body dimensions and simplified trocar positioning. Similar to the significant lower number of conversions in LAP, likely because proper trocar placement is easier due to more surface area and available internal and external space. These findings are preliminary and need to be confirmed in larger studies.

One frequently cited criticism of RAS is its high cost compared to laparoscopy. The SSS^®^ offers notable advantages in this regard: reusable instruments and integration into standard operating rooms can significantly reduce expenses and maintenance costs. This is particularly relevant in pediatric surgery, where case volumes are lower than in adult practice. Conversely, longer operative times, especially during the initial learning phase, may negatively impact cost-effectiveness. Nevertheless, increasing routine and case volume appears to improve efficiency and economic viability over time.

This study has several limitations. Its retrospective design restricts causal inference, and the heterogeneity of procedures (combined with unequal distribution of procedure types across UAP, LAP, TP, and the small-children subgroup) limits direct comparability of results and warrants cautious, descriptive interpretation of subgroup findings. The low number of thoracic operations prevents robust conclusions for this subgroup, despite the relatively higher conversion rate. Additionally, the data were derived from two high-volume centers with substantial expertise in minimally invasive pediatric surgery, which may limit generalizability. Finally, long-term data on functional outcomes, recurrence rates, and growth effects are lacking.

The findings of this study emphasize that RAS in pediatric surgery is a safe alternative to laparoscopy and provide a foundation for further technological advances. Experience with RAS in complex procedures is an essential step toward the development of semi- or fully autonomous robotic systems. In the future, artificial intelligence may handle tasks like camera control or suture assistance. Augmented intelligence will help surgeons in identifying anatomical structures, set no-fly zones, and assisting in intra-operative decisions, aiming to improve precision and lower complication rates. Robotic surgery also expands potential for telesurgery, allowing remote supervision or support of rare procedures by experts.

## Conclusion

Utilization of SSS^®^ for RAS is safe and feasible across the entire pediatric spectrum of surgical procedures and patients, including infants younger than one year and children weighing less than ten kilograms. With increasing experience and further insight into how to utilize and refine RAS, the SSS^®^ has significant potential to advance minimally invasive surgery in children.

## Data Availability

All data are stored securely at the Department of Pediatric Surgery of MosaKids Children’s Hospital/Maastricht University Medical Center+ (MUMC+) and Dr. von Hauner Children’s Hospital of The Ludwig Maximilian University (LMU) University Medical Center of Munich without patient identifiers and is available for inspection upon request.
